# Kinetic nitrogen isotope effects of 18 amino acids degradation during burning processes

**DOI:** 10.1038/s41598-024-65544-w

**Published:** 2024-06-24

**Authors:** Ren-Guo Zhu, Hua-Yun Xiao, Meiju Yin, Hao Xiao, Zhongkui Zhou, Guo Wei, Cheng Liu, Caixia Hu

**Affiliations:** 1 Jiangxi Provincial Key Laboratory of Genesis and Remediation of Groundwater Pollution, East China University of Technology, Nanchang, 330013 China; 2 School of Water Resources and Environmental Engineering, East China University of Technology, Nanchang, 330013 China; 3 School of Agriculture and Biology, Shanghai Jiao Tong University, Shanghai, 200240 China

**Keywords:** Nitrogen isotope effects, Compound-specific nitrogen isotope, Amino acids, Degradation pathways, Biogeochemistry, Environmental sciences

## Abstract

Understanding the nitrogen isotopic variations of individual amino acids (AAs) is essential for utilizing the nitrogen isotope values of individual amino acids (δ^15^N-AA) as source indicators to identify proteinaceous matter originating from biomass combustion processes. However, the nitrogen isotope effects (ε) associated with the degradation of individual amino acids during combustion processes have not been previously explored. In this study, we measured the nitrogen isotope values of residual free amino acids -following a series of controlled combustion experiments at temperatures of 160–240 °C and durations of 2 min to 8 h, as described in Part 1. δ^15^N values of proline, aspartate, alanine, valine, glycine, leucine, and isoleucine are more positive than their initial δ^15^N values after prolonged combustion. Variations in δ^15^N values of the most AAs conform to the Rayleigh fractionation during combustion and their nitrogen isotope effects (ε) are greatly impacted by their respective combustion degradation pathways. This is the first time the ε values associated with the degradation pathways of AAs during combustion have been characterized. Only the ε values associated with Pathway 1 (dehydration to form dipeptide) and 2 (simultaneous deamination and decarboxylation) are found to be significant and temperature-dependent, ranging from + 2.9 to 6.4‰ and + 0.9‰ to + 3.8‰, respectively. Conversely, ε values associated with other pathways are minor. This improves the current understanding on the degradation mechanisms of protein nitrogen during biomass burning.

## Introduction

Several reactive nitrogen species (including NOx, NH_3_, HCN, HNCO, HONO, CH_3_CN and nitrogen-containing organic compounds etc.) are emitted from the burning of vegetation, which are important for atmospheric oxidation processes, atmospheric chemistry, radiative balance and human health^1–4^. Besides that, approximately 50–68% of the fuel nitrogen can be converted to N_2_ and N_2_O during burning, leading to an estimated annual loss of biomass nitrogen (‘pyrodenitrification’), greatly impacting biogeochemical nitrogen cycle of ecosystem^2,5^. These nitrogen-containing species released from biomass burning are from the N-conversion of biomass nitrogen, and nitrogen in biomass predominantly exists in the form of proteins and free amino acids, with a minor proportion present in nucleic acids, chlorophyll, enzymes, vitamins, and alkaloids. Typically, protein-bound nitrogen constitutes approximately 80–85% of the total nitrogen content in biomass^5,6^. The selective conversion of biomass nitrogen into reactive nitrogen or N_2_ and N_2_O are determined by the amino acid compositions of protein in biomass and combustion condition^5,7^. Since the protein composition is very complex and varies greatly from one biomass species to another, fundamental building block of proteins, amino acids (AAs), have been chosen as the model compounds to investigate behavior of biomass nitrogen during combustion processes^6,7^.

Stable nitrogen isotope (δ^15^N) technology serves as a robust tool for identifying or quantifying sources and processes of nitrogen containing molecules within the environment^8,9^. Turekian et al.^10^ attributed the ^15^N enrichment of bulk nitrogen of vegetations during burning to the losses of ^15^N depleted nitrogen resulting from deamination of amino acids. Obviously, compared to δ^15^N values of bulk nitrogen, the nitrogen fraction of individual AAs has great potential to provide more information on the degradation mechanisms of protein nitrogen in the initial stages of biomass burning.

Amino acids, as important atmospheric organic nitrogen compounds, have been found in particles released from biomass burning^11^. Since the δ^15^N values of individual amino acid (δ^15^N-AA) can offer valuable insights into degradation and cycling mechanisms of dissolved organic nitrogen (DON) and particulate organic nitrogen (PON)^8,12–14^, and has been successfully used in ecosystem trophic dynamics^15,16^, biogeochemical nitrogen cycling^12,17^ and paleo-records^18–20^. Unfortunately, studies on variation in δ^15^N-AA values associated with combustion processes are limited. In our prior study, we recorded significantly positive δ^15^N values of glycine (δ^15^N-Gly) in the aerosol particles from biomass burning, exceeding the values from natural sources by up to 21.6‰^21,22^. It is evident that the combustion process can cause substantial nitrogen isotope fractionation of Gly. However, variation in δ^15^N values of other individual amino acids during combustion remain unexplored.

Previous studies have indicated that the initial degradation pathways of AAs during the combustion process primarily include: dehydration reactions, decarboxylation, deamination, concurrent deamination and decarboxylation reactions, self-cyclization dimerization reactions, and hydroxyl substitution of the amide groups (Table 1)^23–31^. It has been demonstrated that amino acids undergo decomposition rather than melting or sublimation processes within the temperature range of 160–240 °C^27^. However, the kinetic isotope effects (ε) associated with the specific degradation pathway of AAs during combustion are still unknown. Moreover, the factors that influence the ε values of individual AAs during burning, such as degradation pathways and combustion temperature, are not well understood.

**Table 1:** The degradation pathways of amino acids during the combustion processes.

	Pathway 1
	Pathway 2
	Pathway 3
	Pathway 4
	Pathway 5
	Pathway 6
	Pathway 7

In this study, we conducted controlled experimental burning of free amino acids in air at temperatures ranging from 160 to 240 °C. The δ^15^N values of individual residual amino acids were determined at set burning temperatures and durations. This study aimed to: first, explore the influence of the burning temperature and time on the δ^15^N-AA values of 18 amino acids; second, determine the ε values associated with the specific degradation pathway of individual amino acids during combustion and analyze the factors influencing ε values.

## Materials and methods

### Combustion experiment of 18 amino acid mixtures

Before the burning experiment, a number of standard mixtures containing the 18 amino acids (Ala (alanine), Asn (asparagine), Asp (aspartate), His (histidine), Gln (glutamine), Glu (glutamate), Gly (glycine), Ile (isoleucine), Pro (proline), Phe (phenylalanine), Leu (leucine), Lys (lysine), Met (methionine), Ser (serine), Thr (threonine), Val (valine), Tyr (tyrosine) and Trp (tryptophan), Sigma-Aldrich, St Louis, MO, USA) each at an equivalent of 1 Mmol was prepared. Then, these were transferred into pre-ashed quartz glass tubes and freeze-dried to solid powder. Time series combustion experiments were constructed at a given temperature (160 °C, 180 °C, 200 °C, 220 °C, and 240 °C, respectively) for varying durations, as detailed in Part 1. To mimic the natural oxic condition during biomass combustion, the experiments are conducted at atmospheric pressure with air as the oxidizing medium. Given the varying decomposition rates of amino acids at different temperatures, the fraction of degraded amino acids increased with the temperature. More AAs degrade at higher temperatures under the same burning duration. To obtain more accurate isotopic fractionation curves of amino acid degradation during the burning, we adjusted the heating duration to be longer at lower temperatures and shorter at higher temperatures. Combustion experiments with the same duration at each given temperature were conducted three times.

### Measurement of δ^15^N values of AAs

After combustion, 250 μL of α-aminobutyric acid (250 μL, 1 nmol μL^−1^) was added into the quartz tube as an internal reference (δ^15^N = − 8.17 ± 0.03‰). Subsequently, the samples were then frozen dried and prepared as AA-tert-butyl dimethylsilyl (tBDMS) derivatives following the method described by our previous study^32,33^. Briefly, approximately 150 μg of anhydrous Na_2_SO_4_, 200 μL of pyridine and 50 μL of N-methyl-N-(tert-butyldimethylsilyl) trifluoroacetamide (MTBSTFA) were added to freeze dried amino acids in sequence. The vials were incubated at 70 °C for 1 h.

δ^15^N values of AA-tert-butyl dimethylsilyl (tBDMS) derivatives were determined by using a Gas Chromatograph(Trace 1310, Thermo Scientific, Bremen, Germany) and an interface (GC IsoLink II + ConFlo IV, Thermo Scientific, Bremen, Germany) connected into an Isotope Ratio Mass Spectrometry (Delta V Advantage, Thermo Scientific, Bremen, Germany). Each reported δ^15^N value represents an average of at least three δ^15^N determinations. For more details of chromatography condition and method validation were provided in supporting information were provided in supporting information.

### Data analysis

All statistical procedures and graphs were conducted using Origin 2021 (OriginLab Corporation, Massachusetts, USA). The structure of amino acids was drawn by Chemoffice 22.0. Data processing involved a nonlinear curve fit to establish a logarithmic regression of δ^15^N values for each residual amino acids at a certain temperature as a function of the ratio of the molar concentration of residual amino acids to their initial concentration (f). The results were accepted for *p* values < 0.05. For a specific amino acid, if the f value at the longest burning time remained above 0.5, indicating the majority of the amino acid remained undegraded, an extrapolation method was employed. This method predicted the nitrogen isotopic fractionation factor (ε) by extrapolating f value to 0. The predictions were accepted if the 95% confidence intervals for ε were less than 2‰ (refer to Tables S2–S6).

## Results

### The offset between the δ^15^N values of individual amino acids pre- and post-combustion

**Figure 1:**
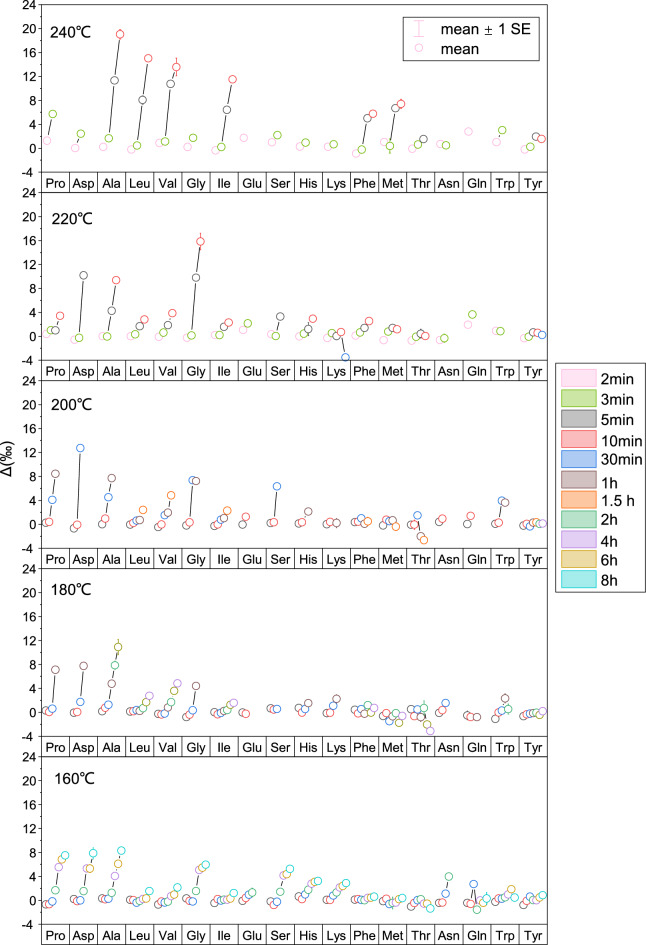
Shifts in δ^15^N values (Δ value) of individual amino acids, comparing the initial values with those after a specified duration of combustion across all experimental temperatures ranging from 160 to 240 °C. The symbols represent the average values.

Figure 1 displays the offset in nitrogen isotope values (Δ value) of 18 amino acids pre- and post- burning at various durations under temperatures of 160 °C, 180 °C, 200 °C, 220 °C, and 240 °C. The results show that under the experimental conditions, the Δ values for the most amino acids exceed 0‰, except for Thr and Gln at 160 °C, Met and Thr at 180 °C, Thr at 200 °C, as well as Lys at 240 °C, which range from − 4‰ to 0‰. The Δ value of individual amino acids is influenced by the combustion duration, combustion temperature, and amino acid type.

#### Influence of combustion duration

With prolonged combustion duration at a consistent temperature, the Δ values of amino acids generally exhibit an increasing trend (Fig. 1). Specifically, at160 °C, the Δ values of Pro, Asp, Ala, and Gly exhibit a sharp increase after a 2-h duration. For instance, the Δ value for Ala remains below 2‰ within 2 h of combustion, but reaching 4.1‰ and 8.3‰ after 4 h and 8 h, respectively. At 180 °C, only combustion for 1 h can significantly increase the Δ values of the above-mentioned four amino acids to be 7.1‰, 7.8‰, 4.8‰, and 4.4‰ respectively. After 3 h, the Δ value of Ala reaches 10.9‰. At 200 °C, a combustion time as short as 30 min facilitate a rapid increase in the Δ values of Pro, Asp, Ala, Gly, and Ser (> 4‰). At 220 °C a substantial Δ value (> 4‰) is observed to Ala only after 5 min combustion, with the Δ values of Asp and Gly even exceeding 8‰. After combustion for 5 min at an even higher temperature of 240 °C, the Δ values of Ala, Leu, Val, and Ile all exceed 8‰ (Asp and Gly were not detected due to complete decomposition during combustion). After 10 min, the Δ values increase further, with Ala’s Δ value approaching 19.0‰ and Leu’s nearing 15.0‰.

#### Influence of combustion temperature

As shown in Fig. 1, the Δ value of amino acids increases with the temperature within a same combustion duration longer than 3 min. After burning for 2 and 3 min at 220 °C and 240 °C, the Δ values of all 18 amino acids remain below 4‰ except for Pro (5.7‰ at 240 °C for 3 min). Though we did not perform combustion experiments for 2 and 3 min at 160 °C, 180 °C, and 200 °C, our experiments conducted for five and ten minutes at these temperatures revealed that the Δ values of all 18 amino acids were less than 2‰. Therefore, it is deduced that combustion for a time shorter than 5 min at these three temperatures would not induce significant alterations in the Δ values of amino acids. At higher temperatures (220 °C and 240 °C), however, a combustion period of 5 min triggers a substantial increase in the Δ values of numerous amino acids. For example, after combustion for 5 min at 220 °C, the Δ values of Asp and Gly approach 10‰; after combustion for 5 min at 240 °C, the Δ values of Ala, Leu, Val, Ile, Phe, and Met increase significantly, with the Δ values of Ala, Leu, and Val exceeding 8‰. If the combustion time is extended to 10 min or longer, these Δ values will continue to increase.

#### Influence of amino acid type

Our combustion experiments indicated that the nitrogen isotope values of seven amino acids—Pro, Asp, Ala, Val, Gly, Leu, and Ile—were markedly influenced by combustion, particularly for Asp, Ala, and Gly (Fig. 1). For instance, following a ten-minute combustion period at 160 °C, 180 °C, 200 °C, 220 °C, and 240 °C, the Δ value of Ala increased rapidly, to 0.2‰, 0.8‰, 1.0‰, 9.4‰, and 19.0‰ respectively. Although the Δ values of Leu, Val, and Ile reached 12‰ after a 10-min combustion at 240 °C, these values remained relatively small (below 4‰) within the temperature range of 160–220 °C.

For other eleven amino acids (Phe, Met, Thr, Trp, Tyr, Ser, Lys, His, Glu, Gln, and Asn), as the burning duration increases, their Δ values show no considerable increase or much lower than that of the aforementioned seven amino acids (Fig. 1). The alterations in nitrogen isotopes of amino acids during combustion are collectively influenced by both the combustion conditions and the amino acid structures. The Δ values of Lys and His, two long-chain poly-nitrogenous amino acids, barely increase with an increase in combustion duration across all experimental temperatures. Specifically, the maximum Δ value of Lys is less than 0.7‰ within the temperature range of 200–240 °C, and the maximum Δ value of His is only 0.9‰ at 240℃. For amino acids with special functional groups (thiol and hydroxyl), such as Thr, Met and Ser, their Δ values display marginal variation. For example, all the Δ values of Thr are less than 1.5‰ during combustion at 160–240 °C across all experimental durations. Barring the high Δ value (7.4‰) of Met at 240 °C, its Δ values at other experimental temperatures remain unaffected by temperature and all stay under 1.1‰. Regarding Ser, aside from elevated Δ values observed at 160 °C and 200 °C after combustion durations of 4 h and 30 min respectively, the changes in its Δ values are not significant at 180 °C, 220 °C and 240 °C, with maximum Δ values below 3.3‰. For amino acids containing a phenyl ring (Tyr, Phe and Trp), the mean Δ value of Tyr at any combustion duration between 160 and 240 °C is less than 2‰. The Δ value of Phe remains constant regardless of the combustion duration within the temperature range of 160–200 °C, with its maximum Δ value staying below 0.7‰. Only at high temperatures (220 °C and 240 °C), its Δ value slightly increases with burning duration, reaching maximum Δ values of 2.5‰ and 5.8‰ at 220 °C and 240 °C, respectively. When Trp is burned at temperatures below 200 °C, the change in its Δ value with burning duration is also negligible, with a maximum Δ value less than 0.8‰. Only at high temperatures is there a slight increase in Trp’s Δ value in conjunction with long combustion time, reaching maximum Δ values of 4.0‰ and 3.0‰ at 200 °C and 240 °C, respectively.

Our results demonstrated that with an increase in combustion temperature and duration, significant increases in the Δ values were only observed for seven amino acids, including Pro, Asp, Ala, Val, Gly, Leu, and Ile. This implies that the combustion process induces a substantial positive shift in the nitrogen isotope values of these seven amino acids.

### Kinetic isotope effects

To further explore the nitrogen isotope fractionation of amino acids during combustion, we analyzed the changes in the nitrogen isotope values (δ^15^N_R_) of residual amino acids across five experimental combustion temperatures in relation to the ratio (f) of the molar concentration of residual amino acids ([AA]_t_) to their initial concentration ([AA]_0_). Note that the combustion reaction process goes from the initial moment f = 1 (all reactants) to the completion of amino acid degradation at f = 0 (all products). f is defined as the fraction of reactant which remains unutilized. Take Asp at 160 °C as an example, its nitrogen isotope value increases from the initial value (δ^15^N_Asp0_) of − 2.6‰ at f = 1 (100% undegraded) to 7.1‰ (δ^15^N_Asp_) at f = 0.06 (6% undegraded). Throughout the burning process, the nitrogen isotope of Asp exhibits a monotonic and concave up increase with the advancement of the reaction, which are consistent with Rayleigh fractionation process (Eq. 1).

1 $$\updelta ^{{{15}}} {\text{N}}_{{\text{R}}} =\updelta ^{{{15}}} {\text{N}}_{{{\text{R}}0}} -\upvarepsilon \times {\text{ln}}\left( {\text{f}} \right)$$

**Figure 2:**
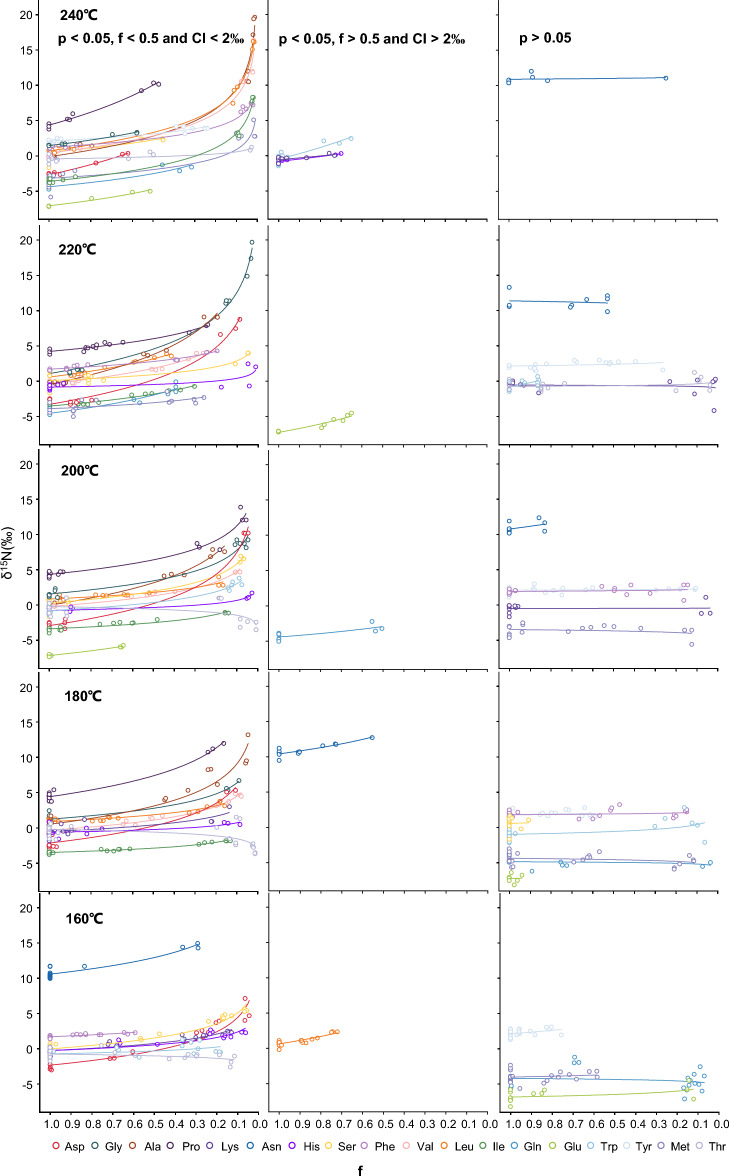
Compound-specific nitrogen isotope compositions of residual amino acids (δ^15^NAA) as a function of its f values during the combustion processes at experimental temperature. δ^15^N_AA_ are linear correlated with ln(f) rather than f (Eq. 1). The slopes of regression lines between δ^15^N_AA_ and ln(f) represent nitrogen isotope effects (ε) for individual amino acids.

The slope (ε) of this equation curve represents the nitrogen kinetic isotope effect of a specific amino acid at a given combustion temperature, and the intercept (δ^15^N_R0_) represents the initial nitrogen isotope value of this amino acid. Rayleigh plots of δ^15^N_R_ for each amino acid after burning at 160 °C, 180 °C, 200 °C, 220 °C and 240 °C are displayed in Fig. 2. We found that in the combustion experiments, variations in nitrogen isotope of the most amino acids conform to the Rayleigh fractionation equation (Eq. 1, Tables S1–S5). However, a few amino acids do not adhere (*p* > 0.05), typically falling into one of two categories:

The first case is that there’s no significant linear correlation between the δ^15^N_R_ and ln(f). Take Glu burned at 160 °C as an example, the initial nitrogen isotope value of Glu is − 7.1‰ ± 0.05‰. Within f range from 0.0 to 0.9, its nitrogen isotope value changes slightly (essentially a horizontal line), ranging from − 8.2 to − 4.5‰ (right column in Fig. 2). Moreover, there is no significant linear relationship (*p* > 0.05) between the nitrogen isotope value (δ^15^N_Glu_) of the residual Glu after burning and its ln(f), indicating that the nitrogen isotope alteration of Glu during the combustion process at 160 °C does not follow the Rayleigh fractionation equation. Similar to Glu, the nitrogen isotope values of residual Gln, Tyr and Met post-combustion for a certain duration at 160 °C do not show a positive linear relationship with their ln(f) (*p* > 0.05). Therefore, we are unable to obtain the nitrogen kinetic isotope effects (ε) of these amino acids during combustion at 160 °C (Table 2).

**Table 2:** The fraction of amino acids which remains unutilized (f) and nitrogen isotope effects (ε, ‰) for individual amino acids degradation during the combustion processes across all experimental temperature range of 160–240 °C.

AAs	160 °C		180 °C		200 °C		220 °C		240 °C	
	f	ε	f	ε	f	ε	f	ε	f	ε
Ala	0.27	6.93	0.05	3.72	0.16	4.57	0.19	6.18	0.01	4.21
Gly	0.08	2.83	0.08	2.22	0.05	2.71	0.03	5.05	0.58	3.50
Leu	0.72	/	0.16	1.41	0.17	1.36	0.42	3.76	0.02	3.76
Pro	0.13	4.61	0.16	4.30	0.06	2.99	0.24	2.62	0.47	8.21
Asp	0.04	2.94	0.11	3.51	0.04	4.52	0.09	5.04	0.62	6.42
Asn	0.29	3.42	0.55	/	0.83	-	0.53	-	0.25	-
Val	0.52	3.80	0.08	2.10	0.08	2.16	0.26	3.19	0.02	3.49
Ile	0.67	3.41	0.13	0.90	0.14	1.27	0.31	2.44	0.02	2.96
Glu	0.12	–	0.99	–	0.64	3.02	0.65	/	0.51	3.40
Gln	0.07	–	0.03	–	0.50	/	0.39	3.74	0.32	2.67
His	0.06	1.15	0.08	0.59	0.03	0.62	0.01	0.55	0.7	/
Lys	0.13	1.46	0.14	1.35	0.04	–	0.02	–	0.73	/
Phe	0.59	1.18	0.15	–	0.14	–	0.2	1.59	0.02	1.77
Tyr	0.8	–	0.59	–	0.11	–	0.26	–	0.24	1.46
Trp	0.18	0.60	0.06	–	0.08	1.67	0.86	–	0.65	/
Met	0.58	–	0.11	–	0.12	–	0.26	1.22	0.01	1.77
Ser	0.06	2.22	0.91	–	0.07	2.42	0.05	1.23	0.45	2.40
Thr	0.12	− 0.38	0.01	− 0.59	0.01	− 0.54	0.04	–	0.03	0.44

–represents *p* > 0.05.

/represents that the f value of this amino acid exceeds 0.5 and its 95% confidence interval (CI) of ε is higher than 2‰. The 95% CI of ε for all amino acids with f > 0.5 at given experimental temperatures are provided in Figs. S1–S5.

In the second case, only a small amount of amino acids are degraded during burning. For instance, Leu, due to its slow pyrolysis rate, has a f value of 0.72 even after 8 h of combustion at 160 °C (as seen middle column in Fig. 2 and Table S1). Isotopic changes caused by combustion degradation of 28% of Leu have a relatively small impact on their initial nitrogen values, thus causing significant errors. Considering that the experimental precision for determining the nitrogen isotopes of amino acid monomers is better than 1.3‰, with an accuracy better than 1.5‰, if the confidence interval of the nitrogen kinetic isotope effect of a specific amino acid at a given combustion temperature, obtained through extrapolation, exceeds 2‰, we deem the error too significant, necessitating the discard of the value. Just as illustrated in Fig. S1, the 95% confidence interval (CI) of ε for Leu at 160 °C, computed using the extrapolation method, is 2.4‰, with an error of more than 2‰. Consequently, even at *p* < 0.01, the ε value determined by fitting the Rayleigh fractionation equation may still have a large error. Therefore, when deriving the ε from the slope of the Rayleigh fractionation curve, two requirements must be met: a significant positive linear correlation between the δ^15^N_R_ and its ln(f) value (*p* < 0.01); and when the f-value exceeds 0.5, the 95% CI of ε should be less than 2‰. The 95% CI of ε for all amino acids with f > 0.5 at given experimental temperatures, obtained using the extrapolation method, are presented in Figs. S1–S5. For amino acids that meet these two conditions (left column in Fig. 2), the ε values during the burning process under our experimental conditions are listed in Table 2.

### Factors influencing ε

#### Amino acid types

**Figure 3:**
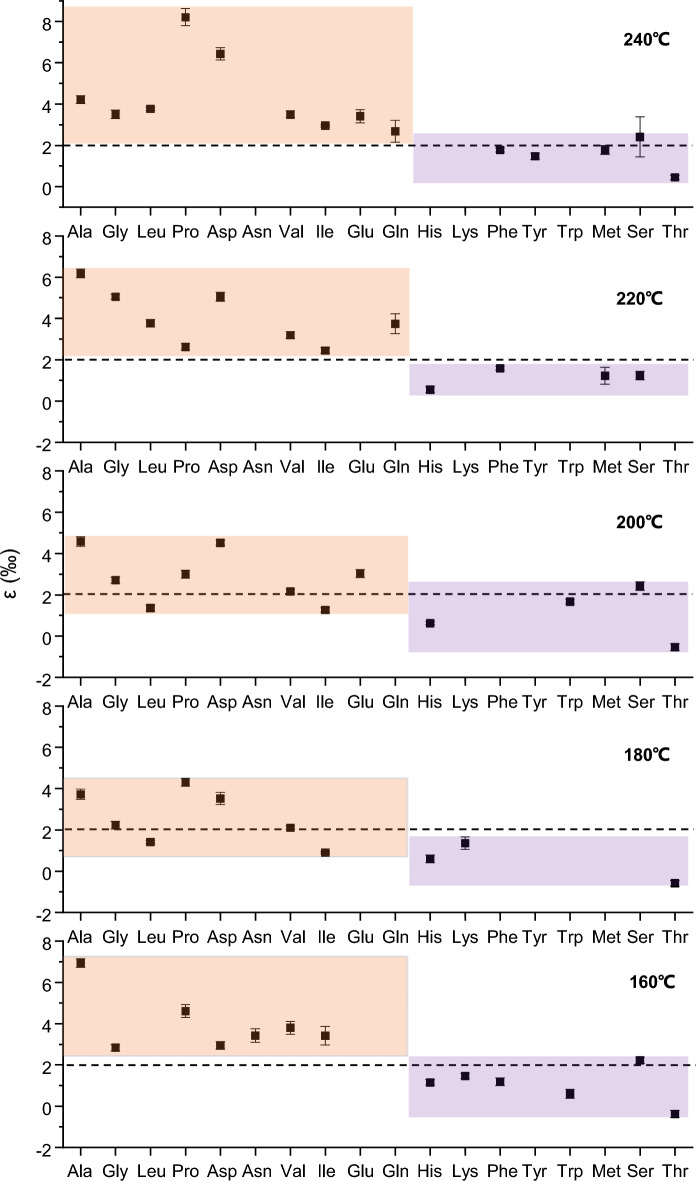
ε (‰) values of individual amino acids at 160 °C, 180 °C, 200 °C, 220 °C and 240 °C, respectively.

Table 2 illustrates large differences in the ε values among the 18 amino acids at experimental combustion temperatures, with Pro, at 240 °C, exhibiting the highest ε value (8.21‰) and the lowest to Thr at 180 °C (− 0.59‰). As shown in Fig. 3, amino acids can be categorized into two categories on the basis of their nitrogen kinetic isotope effects (ε): the first category has ε values typically exceeding 2‰, and shows a substantial degree of variation (1–8‰) corresponding to combustion temperature changes. This category includes Ala, Gly, Leu, Pro, Asp, Asn, Val, Ile, Glu, and Gln (indicated by orange shading in Fig. 3). The second category has smaller ε values, mostly within the 0–2‰ range, and exhibits less variability with temperature changes. This category incorporates His, Lys, Phe, Tyr, Trp, Met, Ser, and Thr (denoted by purple shading in Fig. 3). The observed differences in ε values among 18 amino acids during combustion may be attributable to the amino acids’ structure and distinct combustion mechanisms, which will be further discussed in the subsequent section.

#### Temperature

**Figure 4:**
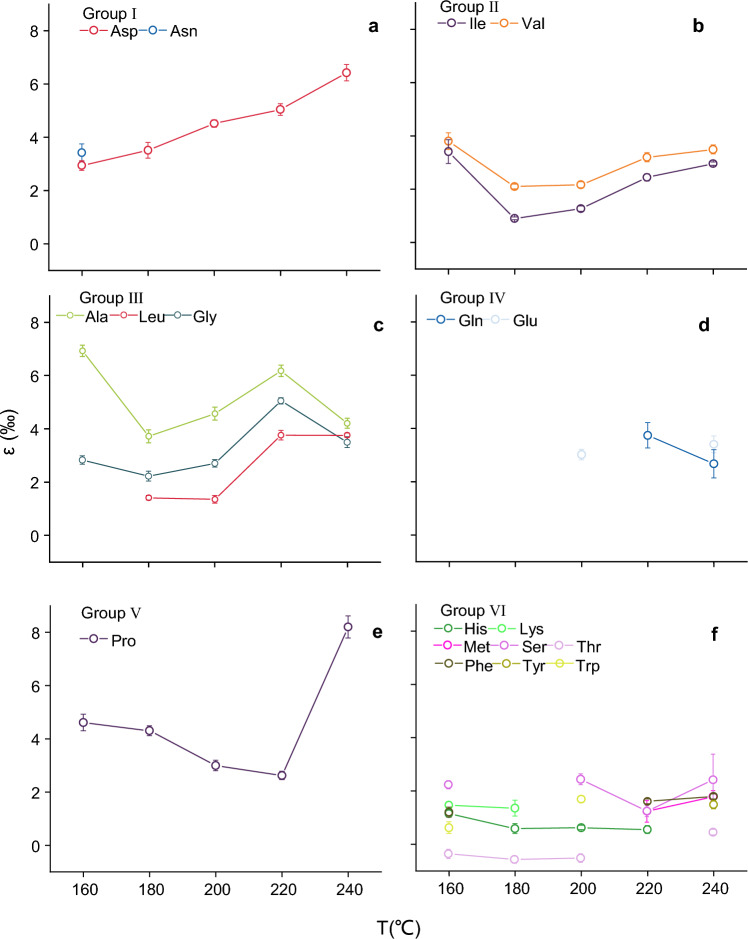
Changes in ε (‰) values of (a) Asp and Asn in group I, (b) Ile and Val in group II, (c) Ala, Leu and Gly in group III, (d) Glu and Gln in group IV, (e) Pro in group V, and (f) His, Lys, Met, Ser, Thr, Phe, Tyr and Trp in group VI with temperature.

Considering the ε values’ variation patterns for individual amino acids at different experimental temperatures, along with their unique structural characteristics, we further divided the amino acids from the first group (marked by orange shading in Fig. 3) into five distinct sub-groups, as depicted in Fig. 4.

Group I comprise two aliphatic chain amino acids: Asn and Asp (Fig. 4a). These two amino acids share similar structures, with Asn being derived from Asp via the substitution of a carboxyl group with an amide group. During combustion, the nitrogen kinetic isotope effect (ε) of Asp and Asn exhibit an upward trend with increasing combustion temperature (Fig. 4a). This trend inversely correlates with the reciprocal of the temperature (1/T) in a linear fashion (1/T) (r^2^ = 0.93, *p* < 0.05) (Fig. S6). The ε values of Asp progressively rise from 2.94‰ at 160 °C to 6.42‰ at 240 °C. For Asn, its ε values initiate at 3.42‰ at 160 °C. Nevertheless, at 180 °C, the isotopic values of Asn conform to the Rayleigh fractionation (*p* < 0.05), the 95% confidence interval for its ε spans from 2.5 to 5.6‰, exceeding 2‰ (Fig. S2). Therefore, we eliminate ε of Asn at 180 °C. When the temperature surpasses 200 °C, the isotopic values of Asn during combustion do not conform to the Rayleigh fractionation process (*p* > 0.05, right column in Fig. 2).

Group II includes two aliphatic branched-chain amino acids, Ile and Val (Fig. 4b). The ε values of these amino acids exhibit a progressive rise with temperature. Additionally, an inverse linear relationship exists between the ε value of Ile and the reciprocal of temperature (1/T), with the slope of this line (8.52) aligning with that of Asp (8.38) (Fig. S6). At 160 °C, Ile records a high ε value (3.41‰, f = 0.67), which drops to 0.9‰ (f = 0.13) as the temperature surpasses 180℃, and subsequently escalates to 2.96‰ (f = 0.02) at 240 °C. Similarly, Val has a large ε value (3.80‰, f = 0.52) at 160 °C, which then drops to 2.1‰ (f = 0.08) at 180 °C, and progressively rises to 3.49‰ (f = 0.02) at 240 °C (Fig. 4b).

Group III comprises three aliphatic branched-chain amino acids: Ala, Leu, and Gly (Fig. 4c). Contrary to group two, the ε values of these three amino acids exhibit wave-like variations with temperature. At 160℃, Ala records an ε value of 6.9‰ (f = 0.27), then decreases to 3.7‰ (f = 0.05) at 180 °C, rises again to 6.2‰ (f = 0.19) at 220 °C, and subtly declines to 4.2‰ (f = 0.01) at 240 °C. The f value of Leu is 0.72, while its 95% CI of ε is above 2‰ at 160 °C, thereby preventing an accurate ε value from being attained. At 180 °C and 200 °C, the ε value of Leu remains stable at 1.4‰, and persists at 3.8‰ at 220 °C and 240 °C. Analogous to Leu, the ε value of Gly is relatively steady between 160 and 200 °C, fluctuating between 2.2‰ and 2.8‰, then rises to 5.0‰ (f = 0.03) at 220 °C, and slightly falls to 3.5‰ (f = 0.58) at 240 °C.

Group IV includes two aliphatic straight-chain amino acids, Glu and Gln (Fig. 4d). Glu and Gln have similar molecular structures, with the side chain carboxyl group of Glu replaced by an amide group to form Gln. When combusted at low temperatures (160–180 °C), the changes in nitrogen isotope values of these two amino acids with the combustion process do not conform to the Rayleigh fractionation (*p* > 0.05) (Fig. 2). The ε values of these two amino acids demonstrate minimal variation of less than 2‰ at higher temperatures (200–240 °C). Specifically, the ε value of Gln decreases slightly from 3.74‰ at 220 °C to 2.67‰ at 240 °C (Fig. 4d). For Glu, the ε value of increases a bit from 3.02‰ at 200 °C to 3.40‰ at 240 °C (Fig. 4d). Although the f values of Gln at 200 °C and of Glu at 220 °C both exceed 0.5 (Table2), the 95% confidence intervals (CI) of their ε values surpass 2‰, suggesting substantial errors in the ε value for Gln at 200 °C and of Glu at 220 °C (Figs. S3 and S4). Consequently, the ε values of Gln at 200 °C and Glu at 220 °C are disregarded.

Group V only includes Pro, which contains a pyrrole ring (Fig. 4e). The ε value of Pro shows a slight decrease from 4.6‰ to 2.6‰ within the temperature range of 160–220 °C, but experiences a substantial increase to 8.2‰ at 240 °C.

All eight amino acids in the second category are divided into Group VI, including three with special functional groups (sulfhydryl and hydroxyl)—Met, Ser, and Thr, three with a phenyl ring—Tyr, Phe, and Trp, and two with multiple nitrogen branched-chain amino acids—His and Lys (Fig. 4f). These eight amino acids exhibit a minor range of ε value changes with temperature.

#### Combustion degradation rate constants

**Figure 5:**
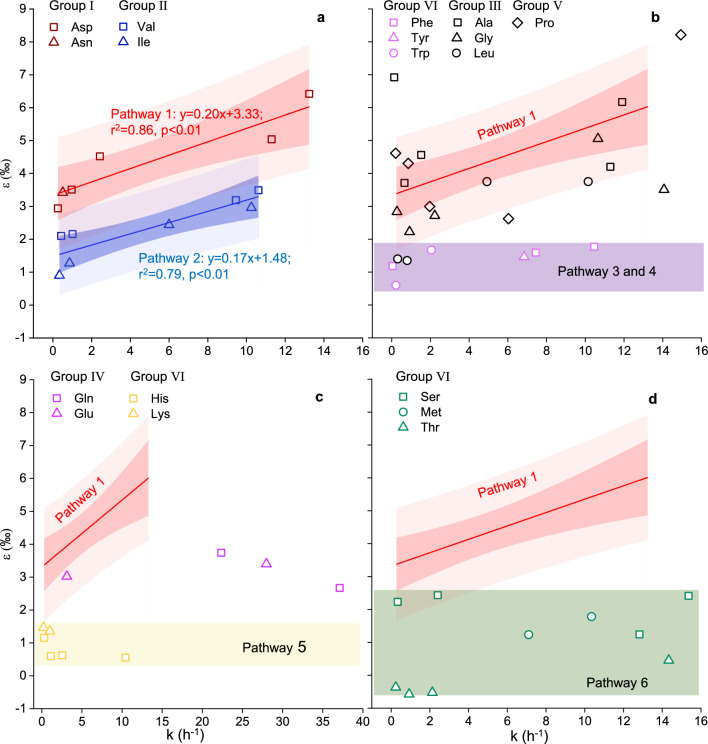
Correlation between ε (‰) values and degradation rate constants (k) for amino acids undergoing degradation via (a) Pathway 1 and Pathway 2, (b) Pathway 3 and Pathway 4, (c) Pathway 5, and (d) Pathway 6.

In the temperature range of 160–240 °C, the nitrogen isotope effects (ε) of Asp and Asn from Group I display a significant positive linear correlation with their respective combustion degradation rate constants during combustion (k) (ε = 0.20 k + 3.33, r^2^ = 0.84, *p* < 0.01), as illustrated by the red line in Fig. 5a. The k values for individual AAs have been referenced from Zhu et al., submitted for publication. Similarly, within the 180–240 °C, the ε values of Ile and Val in Group II also demonstrate a strong positive linear correlation with their k values (ε = 0.17 k + 1.48, r^2^ = 0.79, *p* < 0.01), denoted by the blue line in Fig. 5a. Although the slopes of the red and blue lines are similar, the entirety of Group II is positioned beneath Group I. This suggests that the nitrogen isotope effects of Ile and Val are not as strong as those of Asp and Asn.

The ε values for the three benzene-ring containing amino acids in group VI, Phe, Tyr, and Trp, range between 0.60 and 1.67‰ (as indicated by the purple shadow in Fig. 5b), with only slight variations in relation to their k values. Similarly, the ε values for the two multi-nitrogen amino acids in group VI, His and Lys, range from 0.55 to 1.46‰, and change little with respect to their k values, as depicted by the yellow area in Fig. 5c. Minor fluctuations in the ε values are also observable for the three amino acids that contain unique functional groups, sulfhydryl and hydroxyl, namely Met, Ser, and Thr, with a range of 0.59–2.42‰, which represented by the green area in Fig. 5d.

The ε values for the amino acids in groups III, IV and V do not display a clear association with the reaction rate constant (k). Their ε values fall between the red shadows of group I and the purple or yellow shadows of group VI.

## Discussion

### Impact of the degradation pathway on the nitrogen isotope effects of amino acids

Our results show that the nitrogen isotopic effect of amino acids during the combustion is influenced by the combustion temperature and the type of amino acid. As shown in Fig. 4, the nitrogen isotopic effects of the seven amino acids Asp, Ile, Val, Ala, Leu, Gly, and Pro in Groups I, II, III, and V are larger, and are greatly influenced by the combustion temperature. Conversely, the eleven amino acids Glu, Gln, Phe, Met, Thr, Trp, Tyr, Ser, Lys, His in Groups IV and VI, and Asn in Group I (at low temperatures) undergo negligible nitrogen isotope fractionation during combustion under specific temperature conditions. In addition, some amino acids exhibit nitrogen isotope changes at particular temperatures that do not follow the Rayleigh fractionation processes (right column in Fig. 2 and Table 2). It is widely accepted that variations in temperatures and combustion degradation pathways may result in differences in the nitrogen isotopic effects of amino acids. Previous studies on the reaction mechanisms of amino acids during combustion have shown that the combustion degradation pathways may differ due to the different structures of various types of amino acids. Therefore, amino acids should be categorized by their structures when discussing their respective nitrogen isotopic effects during combustion processes. Given that a single amino acid might follow different combustion degradation pathways at varying combustion temperatures, the impact of the combustion temperature should also be taken into account. Furthermore, some amino acids primarily degrade via a single pathway, whereas others may follow multiple combustion degradation pathways at the same combustion condition, as summarized in Table S6.

**Figure 6:**
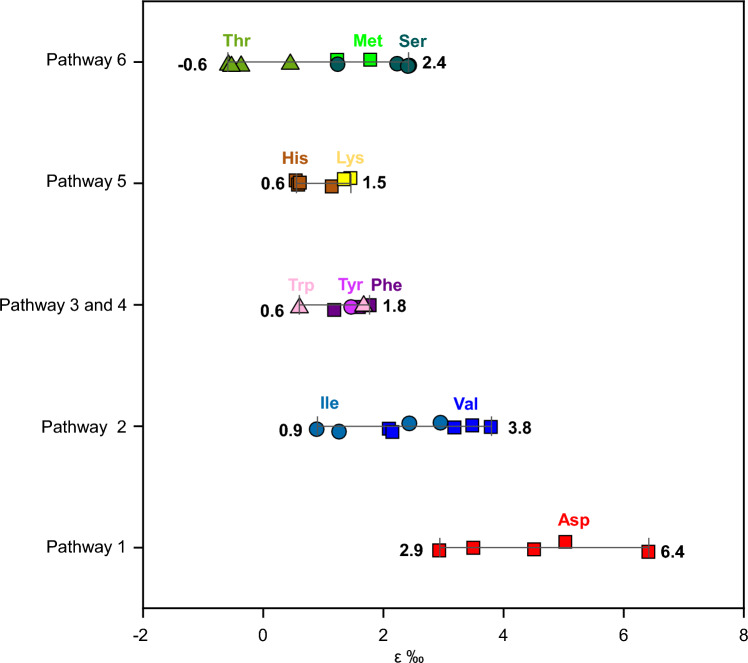
Variation range of ε values with specific degradation pathways of amino acids during the combustion processes. The detailed degradation pathways of amino acids during the combustion processes are list in Table 1.

By discussing the combustion degradation pathways and variations in ε values of individual amino acids with similar structural features across all experimental temperature (Table S6), we have made a preliminary estimate the nitrogen isotope effects (ε value) associated with different degradation pathways of individual amino acids during the combustion processes (Fig. 6).

#### Asp and Asn

Asn and Asp in Group I are two aliphatic chain amino acids. During the combustion process in the range of 160–240 °C, Asp may mainly decompose through Pathway 1, while the decomposition of Asn may be affected by multiple pathways. Sharma et al.^24^ reported that at temperatures less than 300 °C, Asp first forms aspartic acid oligomers (dipeptides or polypeptides) through dehydration condensation (Table S6, Pathway 1), and then decomposes into small molecule nitrogen-containing organic amines or polycyclic aromatic compounds (such as succinimide, trans-succinimide, indole, isoindole dione, naphthalene, and anthracene) through disproportionate and rearrangement^23,27,28^. Similar to Asp, Asn can also form asparagine oligomers through dehydration (Table S6, Pathway 1), and then further decompose to form succinimide and trans-succinimide^28,34^. In addition, Asn can also form aspartic acid oligomers by removing the amide group (Table S6, Pathway 7), followed by further decomposition^27^. After careful comparison of degradation pathways with the nitrogen isotope effects of amino acids at all experimental temperatures, we obtained the ε value of these pathways.

Between 160 and 240 °C, we observed that the ε values associated with the degradation of Asp during the combustion processes, demonstrate a single upward trend with temperature (Fig. 4a) and also exhibit a significantly positive linear correlation with degradation rate constants of Asp (r^2^ = 0.84, *p* < 0.01) (Fig. 5a), thereby confirming that the decomposition of Asp adheres to a single combustion degradation pathway (Pathway 1) within this temperature range, as reported in the literature^24^. For Asn, the ε value at 160 °C closely aligns with that of Asp (Fig. 4a). In conjunction with the ε value of Asp, it exhibits a significant positive linear correlation with the combustion temperature and degradation rate (Fig. 5a and Fig. S1). These suggest that at 160 °C, Asn primarily follows the same combustion degradation pathway (Pathway 1) as Asp, Therefore, it is plausible that the ε range associated with Pathway 1 for Asp, spanning the temperature range of 160–240 °C, and for Asn, within the temperature range of 160–180 °C (Group I), falls between 2.94 to 6.4‰ (Fig. 6).

At 180 °C, the ε value of Asn has a large 95% confidence intervals (CI) and thus is excluded from our consideration (Table S2). At 200 °C and above, nitrogen isotope of Asn no longer adheres to the Rayleigh fractionation model. This can be explained well by multiple pathways (consistent with Pathways 1 and 7 as listed in Table S6) at elevated combustion temperatures.

#### Ile and Val

In Group II, Ile and Val are aliphatic branched-chain amino acids, with their potential combustion degradation pathways listed in Table S6. The primary conversion of these amino acids during combustion involves the formation of dimers via deamination and decarboxylation, as outlined in Pathway 2, followed by subsequent pyrolysis reactions^26^. They believed, post-ionization, the pKa values for Ile’s [M+H]^+^ and CO_2_H+NH_3_^+^ are 2.318 and 9.758, respectively, which are higher than other branched-chain amino acids, including Leu’s 2.328 and 9.744. These figures suggest that the α-CO_2_H and α-NH_2_ groups in Ile exhibit greater acidity and alkalinity compared to other branched-chain amino acids, thereby enhancing Ile’s propensity to form dimers via deamination and decarboxylation (refer to Pathway 2 in Table S6). On the other hand, the α-NH_2_ group in Ile encounters steric hindrance from the alkyl side chain when attempting to attack the central carbon atom in another amino acid molecule. Yet, compared to other branched-chain amino acids like Leu (with a 2-methylpropyl side chain), the 1-methylpropyl side chain in Ile imposes less hindrance on the α-NH_2_ group, thus facilitating its attack. Similar to Ile, Choi and Ko^26^ discovered that Val predominantly forms the dimer 3-methyl-2-(2-methyl propylidene) aminobutyric acid during combustion via deamination and decarboxylation (refer to Pathway 2 in Table S6), proceeded by additional pyrolysis reactions.

In our study, similar to Asp, both Ile and Val exhibited a consistently increasing trend of ε values as the combustion temperature surpassed 180 °C (Fig. 4b). Furthermore, there is a linear positive correlation between the ε values of Ile and Val and their respective degradation rate constants (blue line in Fig. 5a). When combusted at an identical temperature, Ile and Val exhibited similar ε values. These observations suggest that the degradation of Ile and Val during combustion follows the same single pathway (Pathway 2). The ε value associated with Pathway 2 (Group II, as indicated in Fig. 6) is smaller than that of Pathway 1 (Group I, depicted in Fig. 5a), ranging from + 0.9 to + 3.8‰. This explains why, at a same combustion temperature and time, the residual Asp exhibits a larger Δ value than Val and Ile, as displayed in Fig. 1.

At 160 °C, however, both Ile and Val exhibit large ε values, potentially due to these two amino acids degrading through a pathway with a large ε value at low temperatures. Unfortunately, the mechanism underlying this pathway remains to be elucidated.

#### Phe, Tyr and Trp

The potential combustion degradation pathways for Phe, Tyr, and Trp, which all contain aromatic side chains, are outlined in Table S6. Previous research suggests that amino acids with aromatic side chains follow similar pyrolysis pathways during combustion, attributed to the high thermal stability of their aromatic side chains which resist decomposition during combustion^34^. For example, the side chain of Trp is an indole group, which maintains stability at high temperatures. Consequently, Trp predominantly forms tryptamine, 1H-indole, and 3-methyl-1H-indole via decarboxylation (Pathway 3, Table S6) or deamination (Pathway 4, Table S6) during combustion^30,34^. Because the phenyl ring side chain of Phe also remains stable during combustion, it primarily degrades through two pathways: direct decarboxylation to form benzeneethanamine, and simultaneous removal of the amino and carboxyl groups to produce toluene^31^. Tyr, with its phenolic hydroxyl group that also retains stability during combustion, predominantly forms 4-(2-aminoethyl) phenol and 4-methylphenol through decarboxylation or deamination, followed by further decomposition^31^. In this study, the ε values of these three amino acids fell within a narrow range of less than 2‰, significantly smaller than significantly smaller than that of Group I degraded through Pathway 1, Further, their ε values do not vary with temperature, as shown in the purple shaded area in Fig. 5b. Therefore, we believed that the ε values of these amino acids undergoing combustion degradation through Pathways 3 and 4 is relatively small (0.6–1.8‰, Fig. 6) across all the experimental temperatures.

#### His and Lys

Table S6 outlines the potential combustion degradation pathways for two multi-nitrogen amino acids in group VI: His and Lys. Zhao, et al.^30^ found that the degradation of Lys primarily occurs through a self-cyclization pathway (Pathway 5, Table S6) when the temperature is less than 273 °C. They believed that the amino group on the side chain of Lys, –NH_2_, acts as a nucleophile and attacks the carboxyl group, –COOH. This triggers a nucleophilic substitution reaction, resulting in the elimination of a water molecule and forming a seven-membered cyclic amide. This cyclic amide then undergoes further degradation. Similarly, His can form 2-amino-2,4-cyclopentadien-1-one during combustion by linking the main chain C* with C on the imidazole ring, following the self-cyclization pathway^27^. The resultant is a 5-ring connected with the 5-ring of imidazole, and then further decompose^27^. It is clear that during combustion, these two amino acids primarily degrade via Pathway 5. The ε values related to the degradation of these two multi-nitrogen amino acids in this study range from 0.6 to 1.5‰ (Fig. 6). Therefore, we concluded that the nitrogen isotope fractionation effect of the self-cyclization pathway (Pathway 5) for amino acid degradation during combustion is small across all the experimental temperatures (as shown by the yellow shadows in Figs. 4f and 5c).

#### Met, Ser and Thr

There are relatively few reports on the combustion degradation mechanism of three amino acids—Methionine (Met), Serine (Ser), and Threonine (Thr). These amino acids are characterized by unique functional groups (hydroxyl and thiol). Choi and Ko^26^ postulated that during the combustion process, Ser initially forms a dimer (3,6-dihydroxymethyl piperazine-2,5-dione) through dehydration condensation, which then undergoes further degradation. Met is thought to follow a similar pathway, forming a dimer through dehydration condensation before decomposing to yield dimethyl disulfide. The combustion pyrolysis mechanism of Thr remains unclear. The degradation pathway of these amino acids exhibits a small ε value, ranging from − 0.6 to + 2.4‰ across all the experimental temperatures (refer to the green shadow in Figs. 5d and 6). Consequently, in this study, the degradation of these three unique amino acids during combustion is classified separately as Pathway 6.

#### Amino acids degrade via multiple pathways

Unlike those in Groups I, II and VI, amino acids in the other groups—Groups III (Ala, Gly, Leu), IV (Glu, Gln) and V (Pro)—do not fall within the isotopic effect range of the above-mentioned combustion degradation pathway (Figs. 5b, c). The difference may be related to their degradation through multiple combustion pathways.

The nitrogen isotopic values of the residual amino acids degraded through two combustion pathways can be calculated based on the Rayleigh fractionation model and mass balance equation (Eq. 2):

2 $$\updelta ^{{{15}}} {\text{N}} - {\text{AA }} = {\text{f}}_{{\text{i}}} /{\text{f}}*\left( {\updelta ^{{{15}}} {\text{N}} - {\text{AA}}_{0} +\upvarepsilon _{{\text{i}}} *{\text{lnf}}_{{\text{i}}} } \right) \, + {\text{ f}}_{{\text{j}}} /{\text{f}}*\left( {\updelta ^{{{15}}} {\text{N}} - {\text{AA}}_{0} +\upvarepsilon _{{\text{j}}} *{\text{lnf}}_{{\text{j}}} } \right)$$

where f_i_, f_j_, and f represent the molar ratio of residual amino acids after undergoing combustion degradation via Pathway i, Pathway j, and the overall pathway, respectively. Each ratio is evaluated with respect to the initial molar concentration of the amino acids. The δ^15^N-AA_0_ is the nitrogen isotope value of the initial amino acids. The ε_i_ and ε_j_ are nitrogen isotope effects of Pathway i and Pathway j, respectively. If the ε_j_ of Pathway j is very small, i.e. ε_j_ is approximated as 0, Eq. 2 can be approximated as Eq. 3.

3 $$\updelta ^{{{15}}} {\text{N}} - {\text{AA }} =\updelta ^{{{15}}} {\text{N}} - {\text{AA}}_{0} + {\text{f}}_{{\text{i}}} /{\text{f}}*\upvarepsilon _{{\text{i}}} *{\text{lnf}}_{{\text{i}}}$$

Clearly, in the case, the degradation via two pathways still conform to the Rayleigh fractionation model, with a nitrogen isotope effect of f_i_/f*ε_i_. As f_i_/f < 1, the overall nitrogen isotope effect (ε) is smaller than ε_i_. Certainly, if Pathway i contributes the most (f_i_/f is ~ 1), the ε is close to the ε_i_ of Pathway i.

As mentioned above, the nitrogen isotope effects of amino acids degraded via Pathways 3, 4, and 5 are all less than 2‰. Therefore, according to the Eq. 3, if amino acid is degraded simultaneously through Pathways 1 and 3 or 4 or 5, the ε value of the overall degradation processes is approximate to f_1_/f*ε_1_.

Taking Gly as an example, previous research^29,35,36^ has shown that at the temperature of 200–273 °C, Gly is degraded through three pathways, including forming 2,5-diketopiperazine through dehydration condensation (Pathway 1), methylamine through decarboxylation (Pathway 3), and acetic acid through deamination (Pathway 4), with Pathway 1 being the primary degradation pathway. Just as described by Eq. 3, the ε of Gly (black hollow triangles in Fig. 5b) mainly falls within the 95% prediction interval of the nitrogen isotope effect (ε_1_) of Asp, which is degraded only through Pathway 1. Furthermore, similar to Asp, the ε of Gly also increases with its corresponding degradation rate constants, suggesting that Pathway 1 is the main degradation pathway for Gly. At 240 °C, however, the ε value of Gly is below the 95% prediction interval of Pathway 1, yet above the ε ranges of Pathways 3 and 4. This implies that at the temperature Pathways 3 and 4 contribute more to the combustion degradation of Gly. Therefore, the Gly combustion degradation pathways inferred from nitrogen isotope effects are congruent with those identified by chemical methods.

Similar to Gly, Ala and Leu also predominantly degrade via Pathway 1, but also through Pathways 3 and 4^24–26^. The primary degradation pathways for Pro are dehydration condensation (Pathway 1) and decarboxylation (Pathway 3)^25,26^. As these amino acids primarily degrade via Pathway 1 during combustion, the ε values of Ala, Leu, and Pro in this study largely fall within the 95% prediction interval of ε for Pathway 1. Some ε values below the interval are due to the larger contributions of Pathways 3 and 4. Notably, at 160 °C, Ala has a big ε value, higher than 95% prediction interval of ε_1_ of Pathway 1, possibly because that the degradation of Ala at low temperatures is through a pathway with a stronger nitrogen isotope effect.

The degradation pathways for Glu and Gln in during combustion are complex^24^. In addition to forming a dimer via dehydration condensation (Pathway 1) and subsequently dehydrating to form a six-membered ring glutarimide, Glu can also create an intramolecular amide compound, pyroglutamic acid, through the dehydration of an amino group and a carboxyl group (a self-cyclization pathway, Pathway 5). Alternatively, it can form pyrrolidinone through Pathway 5 after decarboxylation at the C2 position (Pathway 3), and then degrade further^23^. Sharma, et al.^24^ discovered that, besides Glu’s aforementioned degradation pathways, Gln can also undergo degrade through the deamination pathway (Pathway 4). Although the ε values of Glu and Gln fall between the 95% prediction interval of Pathway 1 and the range of Pathways 5, ε values of Glu and Gln are more close to the range of the pathways 5, suggesting other Pathways rather than Pathway 1 play a more significant role in the combustion degradation of these two amino acids, as illustrated in Fig. 5c.

### Implication

Prior studies have suggested an inconsistent pattern in the alterations of the isotope composition of bulk nitrogen in organic matter following combustion^37–39^. The isotope values of bulk nitrogen in residual organic matter may exhibit a multitude of variations, leaning towards either positive or negative, or demonstrating minor fluctuations^10^. Turekian et al.^10^ speculated that this variation may be related to the accessing of different nitrogen pools during different levels of heating. The residual material following combustion comprises a mixture of multiple nitrogenous compounds, with only a fraction of the total nitrogenous compounds identified. Since the composition and δ^15^N values of the residual nitrogenous compounds vary with combustion temperature and duration, inconsistent alterations in bulk δ^15^N are observed during combustion. Consequently, the precise estimation of bulk δ^15^N variation during combustion is challenging. Notably, δ^15^N measurements directly on specific amino acids are not influenced by other nitrogen-containing compounds in biomass. Unlike the fluctuations of bulk δ^15^N, the nitrogen isotope of Pro, Asp, Ala, Val, Gly, Leu, and Ile exhibits a consistent and concave increase as the burning process progresses. The nitrogen isotopic fractionation of these AAs during combustion process can lead to marked enrichment of ^15^N compared to their initial values. With an increase in combustion temperature and duration, the offset in nitrogen isotope values of Ala pre- and post- burning can even reach 19‰. Such significant and consistent enrichment in δ^15^N values of these individual amino acids during combustion process can be utilized to trace biomass burning source.

## Conclusions

The nitrogen isotope effects associated with different degradation pathways of individual amino acids during the combustion processes were explored in this study. Our findings reveal that only seven out of 18 amino acids—Asp, Ile, Val, Ala, Leu, Gly, and Pro—undergo significant nitrogen isotopic fractionation (ε > 2‰) during combustion. Other 11 amino acids—Glu, Gln, Phe, Met, Thr, Trp, Tyr, Ser, Lys, His, and Asn—exhibit negligible nitrogen isotope fractionation (ε < 2‰) under the same conditions. The difference in nitrogen isotope effects of these individual amino acids is influenced by their respective combustion degradation pathways and temperature. Except for Pathway 7, the remaining six pathways all conform to the Rayleigh fractionation model. Only Pathways 1 and 2 could induce large nitrogen isotope fractionation of amino acids, while the nitrogen isotope effects associated with Pathways 3, 4, 5, and 6 are small. The ε for the amino acids degraded through Pathways 1 and 2 significantly increases with combustion temperature, also exhibiting a linear positive correlation with their corresponding degradation rate constants, whereas for other degradation pathways, there is not change of the ε with combustion temperature. To determine the nitrogen isotope effects of individual amino acids during combustion processes, temperatures ranging from 160 to 240 °C were employed in this study. The temperature range during field combustion processes would exceed the narrower range utilized in this experiment. Consequently, the primary pathway of amino acid degradation can only be qualitatively assessed based on variations in its compound-specific δ^15^N values when degradation occurs through multiple pathways.

### Supplementary Information

Supplementary Information.

## Data Availability

Data is provided within the manuscript or supplementary information files.
