# Supplementary material for: Kinetic nitrogen isotope effects of 18 amino acids degradation during burning processes
Source: Sci Rep. 2024 Jun 24;14:14559. doi: 10.1038/s41598-024-65544-w (PMC11196629; doi:10.1038/s41598-024-65544-w)
Supplement: Supplementary file 1 — Supplementary Information. [file 41598_2024_65544_MOESM1_ESM.pdf]

Kinetic nitrogen isotope effects of 18 amino acids degradation during burning processes

Ren-Guo Zhu [a,b], Hua-Yun Xiao [c,*], Meiju Yin [c], Hao Xiao [c], Zhongkui Zhou [b], Guo Wei [a,b], Chen Liu [a,b], Caixia Hu [a,b]

[a]Jiangxi Province Key Laboratory of the Causes and Control of Atmospheric Pollution, East China University of Technology, Nanchang 330013, China.

[b]School of Water Resources and Environmental Engineering, East China University of Technology, Nanchang 330013, China.

[c]School of Agriculture and Biology, Shanghai Jiao Tong University, Shanghai 200240, China

[*]Corresponding author: Hua-Yun Xiao (Xiaohuayun@sjtu.edu.cn)

Table of Contents

Chromatography condition and method validation	Page S2
Table S1	Page S3
Table S2	Page S4
Table S3	Page S5
Table S4	Page S6
Table S5	Page S7
Table S6	Page S8
Figure S1	Page S9
Figure S2	Page S10
Figure S3	Page S11
Figure S4	Page S12
Figure S5	Page S13
Figure S6	Page S14
Figure S7	Page S15

Chromatography condition and method validation

The analysis employed a gas chromatography-mass spectrometry-isotope ratio mass spectrometry (GC/MS/IRMS) system, utilizing a DB-5 column (30 m × 0.25 mm × 0.25 μm; Thermo Scientific, Bremen, Germany) for the separation of amino acids. A 1.0 μL sample was injected in splitless mode, with the autosampler injector maintained at 270°C. The helium carrier gas was regulated at a flow rate of 1.0 mL/min. The system was back-flushed with helium for 900s in each sample analysis. The GC oven's temperature program commenced at 90°C (held for 1 minute), then increased at 12°C/min to 150°C (held for 5 minutes), followed by a ramp at 3°C/min to 220°C, and a final ramp at 12°C/min to 285°C (held for 7.5 minutes). The combustion reactor temperature was set at 1,000°C. The analytical run was only accepted if the differences of $\delta^{15}N$ values of the internal reference (α-aminobutyric acid, $\delta^{15}N$=-8.17±0.03‰) between GC-C-IRMS and Elemental Analyzer (Flash 2000, Thermo Scientific, Bremen, Germany)-Isotope Ratio Mass Spectrometry (253 Plus, Thermo Scientific, Bremen, Germany) values were less than ±1.5‰. To monitor the measurement performance and to normalized the $\delta^{15}N$ values of AAs, a standard mixture containing 18 amino acids, with their $\delta^{15}N$ values were individually determined by EA-IRMS, was examined every six samples in each GC-C-IRMS determination sequence. The $\delta^{15}N$ values of the underivatized amino acids measured by EA/IRMS were linearly correlated with those of the derivatized amino acids measured by GC/C/IRMS (R^2=0.997, $P < 0.001$). The variation between the empirically corrected $\delta^{15}N$ values measured by EA/IRMS and GC/MS/IRMS were better than 1.3‰. The analytical precision (SD, n=3) of the $\delta^{15}N$ value was better than 1.4‰.

Table S1. Rayleigh equation (r^2, p and f) and CI values of 18 amino acids at 160°C. f is the residual ratio for the longest burning time (6 hours). CI is 95% confidence interval (s) of ε obtained by extrapolation when the residue ratio is 0.

AAs	Rayleigh equations	r^2	p	f	CI
Ala	y=-6.93*Ln(f)-0.13	0.98	<0.05	0.27	<2‰
Gly	y=-2.83*Ln(f)+1.65	0.94	<0.05	0.08	<2‰
Leu	y=-4.81*Ln(f)+0.63	0.76	<0.05	0.72	2.4‰
Pro	y=-4.61*Ln(f)+4.16	0.91	<0.05	0.13	<2‰
Asp	y=-2.94*Ln(f)-2.32	0.92	<0.05	0.04	<2‰
Asn	y=-3.42*Ln(f)+10.57	0.89	<0.05	0.29	<2‰
Val	y=-3.80*Ln(f)-0.55	0.87	<0.05	0.52	<2‰
Ile	y=-3.41*Ln(f)-3.52	0.71	<0.05	0.67	<2‰
Glu	y=-0.52*Ln(f)-6.85	0.21	>0.05	0.12	-
Gln	y=0.23*Ln(f)-4.19	0.03	>0.05	0.07	-
His	y=-1.15*Ln(f)-0.26	0.79	<0.05	0.06	<2‰
Lys	y=-1.46*Ln(f)-0.27	0.86	<0.05	0.13	<2‰
Phe	y=-1.18*Ln(f)+1.70	0.64	<0.05	0.59	<2‰
Tyr	y=-2.16*Ln(f)+2.13	0.11	>0.05	0.8	-
Trp	y=-0.60*Ln(f)-0.74	0.25	<0.05	0.18	<2‰
Met	y=-0.58*Ln(f)-4.03	-0.02	>0.05	0.58	-
Ser	y=-2.22*Ln(f)-0.02	0.91	<0.05	0.06	<2‰
Thr	y=0.38*Ln(f)-0.75	0.18	<0.05	0.12	<2‰

Table S2. Rayleigh equation (r^2, p and f) and CI values of 18 amino acids at 180°C. f is the residual ratio for the longest burning time (4 hours). CI is 95% confidence interval (s) of ε obtained by extrapolation when the residue ratio is 0.

AAs	Rayleigh equation	r^2	p	f	CI
Ala	y=-3.72*Ln(f)+0.51	0.92	<0.05	0.05	<2‰
Gly	y=-2.22*Ln(f)+1.24	0.91	<0.05	0.08	<2‰
Leu	y=-1.41*Ln(f)+0.88	0.92	<0.05	0.16	<2‰
Pro	y=-4.30*Ln(f)+4.40	0.97	<0.05	0.16	<2‰
Asp	y=-3.51*Ln(f)-2.16	0.92	<0.05	0.11	<2‰
Asn	y=-4.03*Ln(f)+10.41	0.77	<0.05	0.55	3.1‰
Val	y=-2.10*Ln(f)-0.35	0.96	<0.05	0.08	<2‰
Ile	y=-0.90*Ln(f)-3.50	0.93	<0.05	0.13	<2‰
Glu	y=-0.85*Ln(f)-7.24	0.001	p>0.05	0.99	-
Gln	y=0.15*Ln(f)-4.86	-0.05	p>0.05	0.03	-
His	y=-0.59*Ln(f)-0.63	0.44	<0.05	0.08	<2‰
Lys	y=-1.35*Ln(f)-0.52	0.58	<0.05	0.14	<2‰
Phe	y=-0.16*Ln(f)+0.18	0.01	p>0.05	0.15	-
Tyr	y=-0.35*Ln(f)+1.83	-0.03	p>0.05	0.59	-
Trp	y=-0.60*Ln(f)-0.98	0.21	p>0.05	0.06	-
Met	y=0.27*Ln(f)-4.38	0.04	p>0.05	0.11	-
Ser	y=-1.03*Ln(f)+0.56	0.001	p>0.05	0.91	-
Thr	y=0.59*Ln(f)-0.20	0.45	<0.05	0.01	<2‰

Table S3. Rayleigh equation (r^2, p and f) and CI values of 18 amino acids at 200℃. f is the residual ratio for the longest burning time (1.5 hours). CI is 95% confidence interval (s) of ε obtained by extrapolation when the residue ratio is 0.

AAs	200	r^2	p	f	CI
Ala	y=-4.57*Ln(f)-0.025	0.96	<0.05	0.16	<2‰
Gly	y=-2.71*Ln(f)+1.55	0.96	<0.05	0.05	<2‰
Leu	y=-1.36*Ln(f)+0.84	0.84	<0.05	0.17	<2‰
Pro	y=-2.99*Ln(f)+4.32	0.94	<0.05	0.06	<2‰
Asp	y=-4.52*Ln(f)-2.94	0.99	<0.05	0.04	<2‰
Asn	y=-3.70*Ln(f)+10.77	0.17	>0.05	0.83	-
Val	y=-2.16*Ln(f)-0.22	0.96	<0.05	0.08	<2‰
Ile	y=-1.27*Ln(f)-3.39	0.9	<0.05	0.14	<2‰
Glu	y=-3.02*Ln(f)-7.18	0.97	<0.05	0.64	<2‰
Gln	y=-2.16*Ln(f)-4.41	0.62	<0.05	0.5	3.0‰
His	y=-0.62*Ln(f)-0.78	0.85	<0.05	0.03	<2‰
Lys	y=-0.02*Ln(f)-0.52	0.001	>0.05	0.04	-
Phe	y=-0.14*Ln(f)+1.91	0.03	>0.05	0.14	-
Tyr	y=-0.08*Ln(f)+2.10	0.02	>0.05	0.11	-
Trp	y=-1.67*Ln(f)-0.81	0.94	<0.05	0.08	<2‰
Met	y=0.26*Ln(f)-3.49	0.06	>0.05	0.12	-
Ser	y=-2.42*Ln(f)+0.25	0.93	<0.05	0.07	<2‰
Thr	y=0.54*Ln(f)-0.22	0.41	<0.05	0.01	<2‰

Table S4. Rayleigh equation (r^2, p and f) and CI values of 18 amino acids at 220°C. f is the residual ratio for the longest burning time (10min). CI is 95% confidence interval (s) of ε obtained by extrapolation when the residue ratio is 0.

AAs	220	R^2	p	f	CI
Ala	y=-6.18*Ln(f)-0.45	0.98	<0.05	0.19	<2‰
Gly	y=-5.05*Ln(f)+1.07	0.99	<0.05	0.03	<2‰
Leu	y=-3.76*Ln(f)+0.58	0.97	<0.05	0.42	<2‰
Pro	y=-2.62*Ln(f)+4.21	0.95	<0.05	0.24	<2‰
Asp	y=-5.04*Ln(f)-3.27	0.98	<0.05	0.09	<2‰
Asn	y=0.50*Ln(f)+11.37	0.02	>0.05	0.53	-
Val	y=-3.19*Ln(f)-0.37	0.96	<0.05	0.26	<2‰
Ile	y=-2.44*Ln(f)-3.52	0.96	<0.05	0.31	<2‰
Glu	y=-5.38*Ln(f)-7.27	0.88	<0.05	0.65	3.3‰
Gln	y=-3.74*Ln(f)-4.55	0.88	<0.05	0.39	<2‰
His	y=-0.55*Ln(f)-0.84	0.56	<0.05	0.01	<2‰
Lys	y=0.09*Ln(f)-0.56	0.02	>0.05	0.02	-
Phe	y=-1.59*Ln(f)+1.69	0.96	<0.05	0.2	<2‰
Tyr	y=-0.36*Ln(f)+2.13	0.02	>0.05	0.26	-
Trp	y=-5.79*Ln(f)-0.82	0.35	>0.05	0.86	-
Met	y=-1.22*Ln(f)-3.90	0.41	<0.05	0.26	<2‰
Ser	y=-1.23*Ln(f)+0.084	0.76	<0.05	0.05	<2‰
Thr	y=-0.16*Ln(f)-0.76	0.07	p>0.05	0.04	-

Table S5. Rayleigh equation (r^2, p and f) and CI values of 18 amino acids at 240℃. f is the residual ratio for the longest burning time (3min). CI is 95% confidence interval (s) of ε obtained by extrapolation when the residue ratio is 0.

AAs	240	R^2	p	f	CI
Ala	y=-4.21*Ln(f)-0.11	0.97	<0.05	0.01	<2‰
Gly	y=-3.50*Ln(f)+1.45	0.98	<0.05	0.58	<2‰
Leu	y=-3.76*Ln(f)+0.70	0.99	<0.05	0.02	<2‰
Pro	y=-8.21*Ln(f)+4.34	0.98	<0.05	0.47	<2‰
Asp	y=-6.42*Ln(f)-2.68	0.98	<0.05	0.62	<2‰
Asn	y=-0.15*Ln(f)+10.87	0.02	>0.05	0.25	-
Val	y=-3.49*Ln(f)+0.41	0.97	<0.05	0.02	<2‰
Ile	y=-2.96*Ln(f)-3.61	0.99	<0.05	0.02	<2‰
Glu	y=-3.4*Ln(f)-7.08	0.96	<0.05	0.51	<2‰
Gln	y=-2.67*Ln(f)-4.37	0.86	<0.05	0.32	<2‰
His	y=-3.04*Ln(f)-0.81	0.79	<0.05	0.7	2.9‰
Lys	y=-2.41*Ln(f)-0.57	0.56	<0.05	0.73	3.8‰
Phe	y=-1.77*Ln(f)+1.22	0.96	<0.05	0.02	<2‰
Tyr	y=-1.46*Ln(f)+2.09	0.87	<0.05	0.24	<2‰
Trp	y=-7.30*Ln(f)-0.48	0.78	<0.05	0.65	6.8‰
Met	y=-1.77*Ln(f)-3.18	0.84	<0.05	0.01	<2‰
Ser	y=-2.40*Ln(f)+0.58	0.47	<0.05	0.45	<2‰
Thr	y=-0.44*Ln(f)-0.45	0.65	<0.05	0.03	<2‰

Table S6. The structural characteristics and respective degradation pathways of individual amino acids as delineated in the literature.

Group	AAs	Structure	Pathway	References
I	Asp	aliphatic amino acid	One	[1-4]
	Asn	aliphatic amino acid	One, Seven	[1,2,5]
II	Ile	aliphatic amino acid	Two	[6]
	Val	aliphatic amino acid	Two	[6]
III	Ala	aliphatic amino acid	One, three, Four	[6]
	Gly	aliphatic amino acid	One, three, Four	[7-9]
	Leu	aliphatic amino acid	One, three, Four	[6,10]
IV	Glu	aliphatic amino acid	One, three, Five	[1-4,11]
	Gln	aliphatic amino acid	One, Four, Five	[1,2]
V	Pro	Pyrrole cyclic amino acid	One, Three	[6]
VI	Phe	Phenyl-containing amino acid	Three, Four	[12]
	Trp	Phenyl-containing amino acid	Three, Four	[5,13]
	Tyr	Imidazole ring amino acids	Three, Four	[12]
	His	Polynitrogen amino acid	Five	[1]
	Lys	Polynitrogen amino acid	Five	[13,14]
	Met	sulfhydryl amino acids	Six	[6]
	Ser	Hydroxyamino acid	Six	[6]
	Thr	Hydroxyamino acid	Six	-

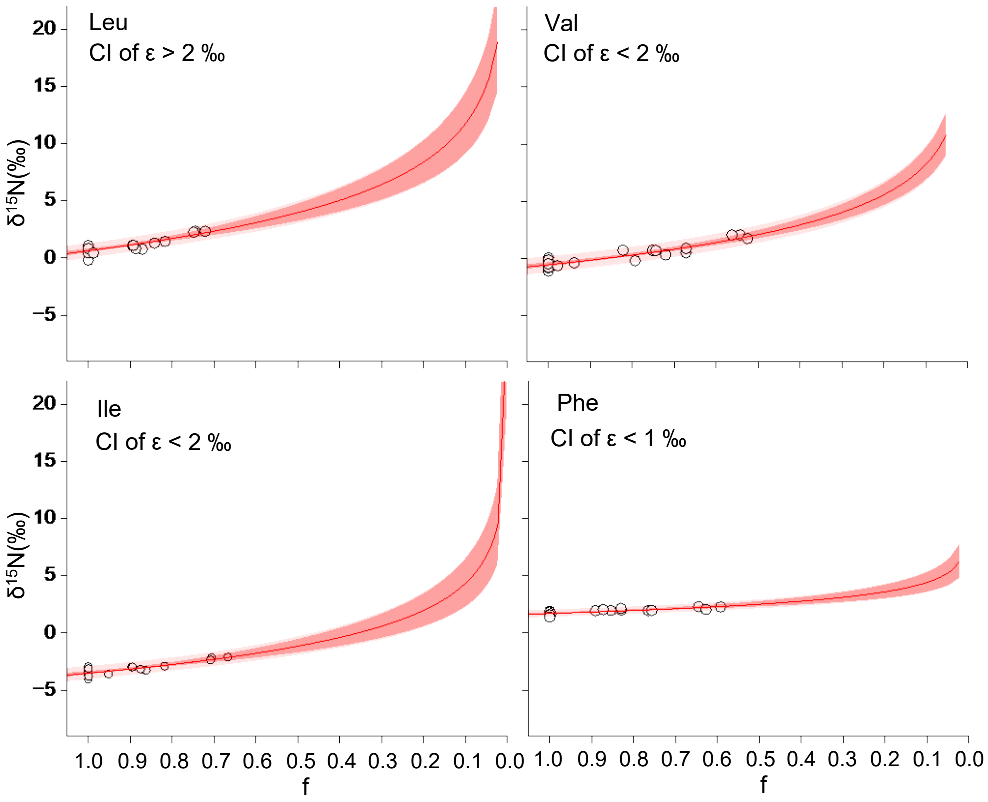

Figure S1. 95% confidence intervals of four AAs by extrapolation to the residue ratio (f) of 0 at 160°C. The f value of the longest burning time for the four AAs are more than 0.5, while for the other AAs with p values below 0.05, they are less than 0.5 and their 95% confidence intervals of ε are less than 2‰ (listing in Table S1).

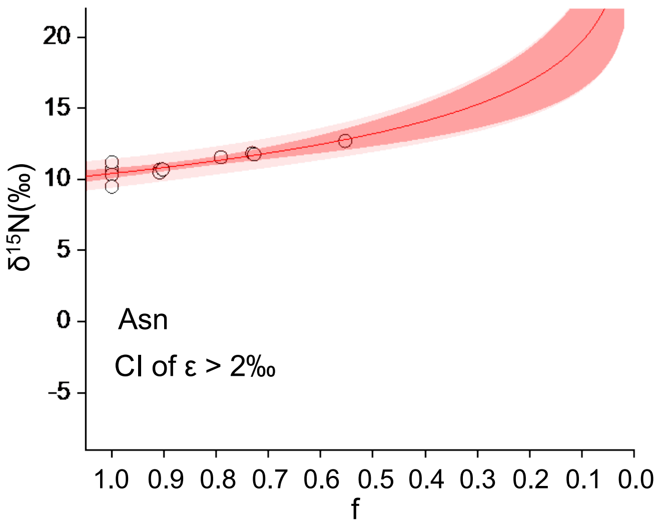

Figure S2. 95% confidence interval of asparagine by extrapolation to the residue ratio (f) of 0 at 180°C. The f value of the longest burning time for the asparagine is more than 0.5, while for the other AAs with p values below 0.05, they are less than 0.5 and their 95% confidence intervals of ε are less than 2‰ (listing Table S2).

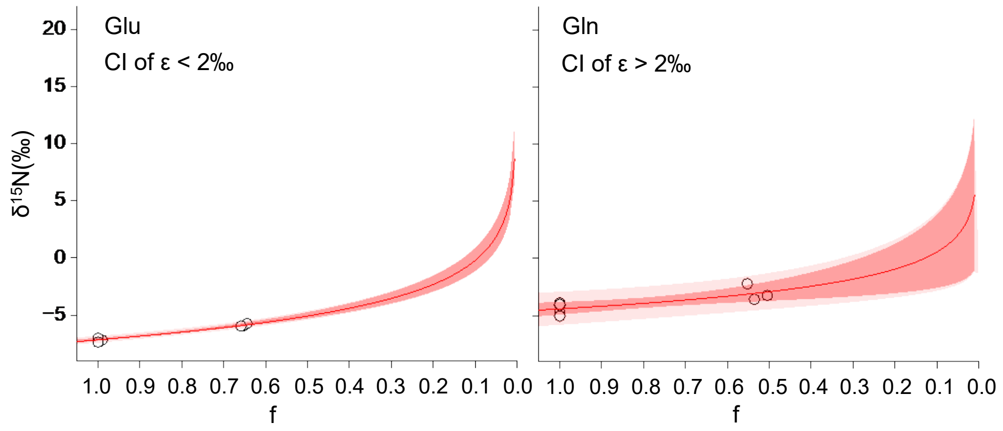

Figure S3. 95% confidence intervals of glutamic acid and glutamine by extrapolation to the residue ratio (f) of 0 at 200°C. The f value of the longest burning time for the glutamic acid and glutamine are more than 0.5, while for the other AAs with p values below 0.05, they are less than 0.5 and their 95% confidence intervals of ε are less than 2‰ (listing in Table S2).

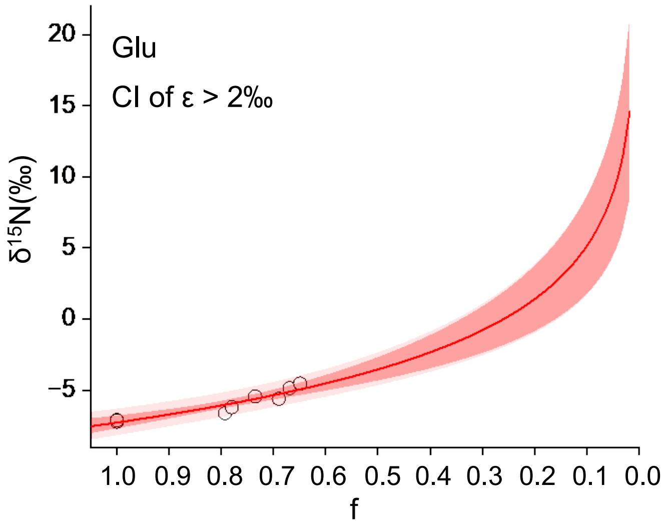

Figure S4. 95% confidence interval of glutamic acid by extrapolation to the residue ratio (f) of 0 at 220°C. The f value of the longest burning time for the glutamic acid is more than 0.5, while for the other AAs with p values below 0.05, they are less than 0.5 and their 95% confidence intervals of ε are less than 2‰ (listing in Table S2).

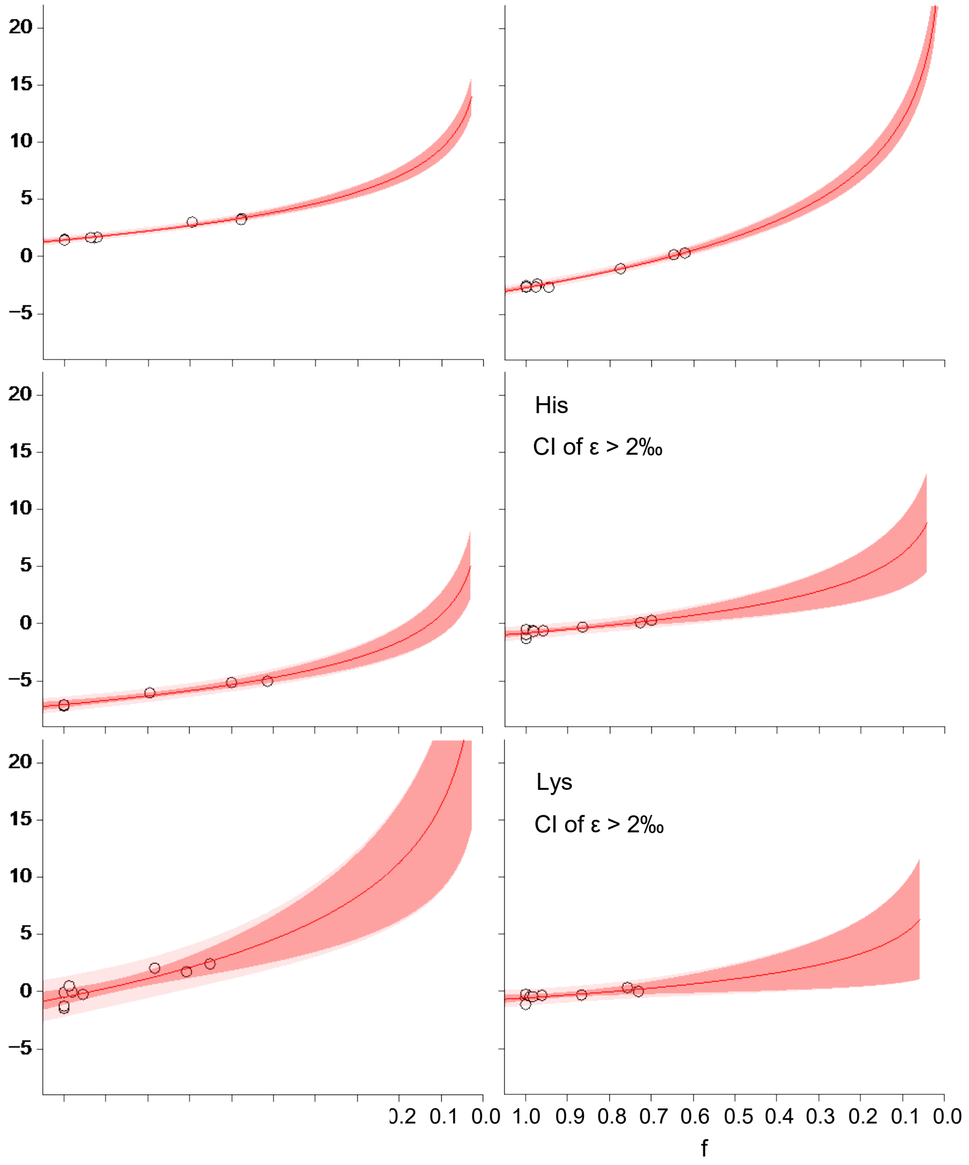

Figure S5. 95% confidence interval of the six AAs by extrapolation to the residue ratio (f) of 0 at 240℃. The f value of the longest burning time for the six AAs are more than 0.5, while for the other AAs with p values below 0.05, they are less than 0.5 and their 95% confidence intervals of ε are less than 2‰ (listing Table S2).

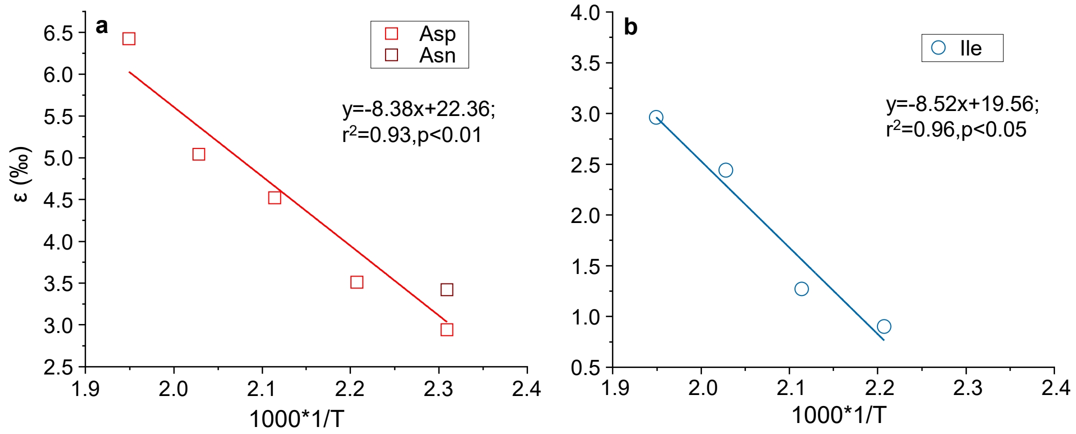

Figure S6. Correlations of the ε values of Asp and Asn (a) and Ile (b) with 1000*1/T. T is the burning temperature (K).

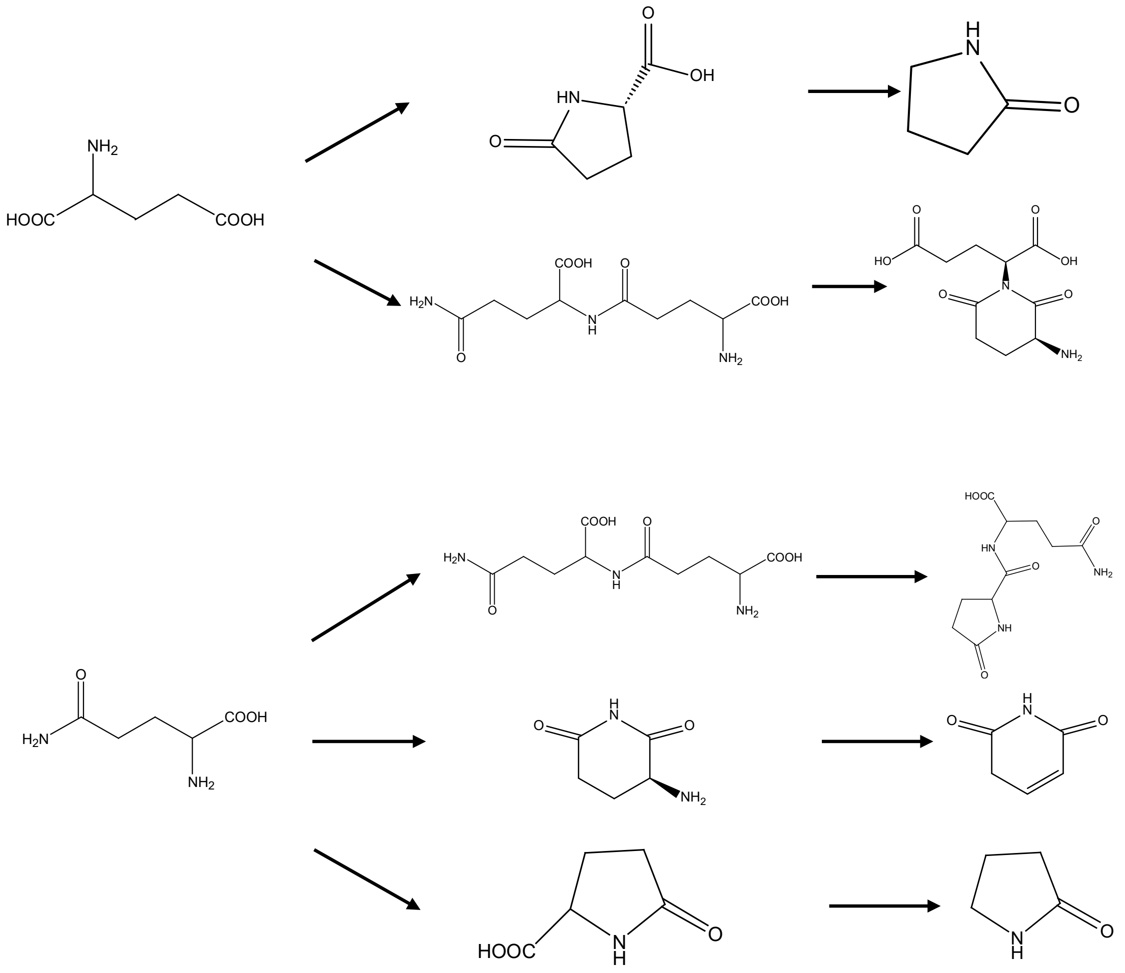

Figure S7. The major degradation pathways for Glu and Gln during the combustion process.

References

1 Weiss, I. M., Muth, C., Drumm, R. & Kirchner, H. O. K. Thermal decomposition of the amino acids glycine, cysteine, aspartic acid, asparagine, glutamic acid, glutamine, arginine and histidine. *BMC Biophys* **11**, 2, https://doi.org/10.1186/s13628-018-0042-4 (2018).

2 Sharma, R. K. *et al.* On the role of peptides in the pyrolysis of amino acids. *J. Anal. Appl. Pyrolysis* **72**, 153-163, https://doi.org/10.1016/j.jaap.2004.03.009 (2004).

3 Sharma, R. K., Chan, W. G. & Hajaligol, M. R. Product compositions from pyrolysis of some aliphatic α-amino acids. *J. Anal. Appl. Pyrolysis* **75**, 69-81, https://doi.org/10.1016/j.jaap.2005.03.010 (2006).

4 Jiang, D. *et al.* Cyclic Compound Formation Mechanisms during Pyrolysis of Typical Aliphatic Acidic Amino Acids. *ACS Sustain. Chem. Eng.* **8**, 16968-16978, https://doi.org/10.1021/acssuschemeng.0c07108 (2020).

5 Sharma, R. K., Chan, W. G., Seeman, J. I. & Hajaligol, M. R. Formation of low molecular weight heterocycles and polycyclic aromatic compounds (PACs) in the pyrolysis of α-amino acids. *J. Anal. Appl. Pyrolysis* **66**, 97-121, https://doi.org/10.1016/S0165-2370(02)00108-0 (2003).

6 Choi, S.-S. & Ko, J.-E. Dimerization reactions of amino acids by pyrolysis. *J. Anal. Appl. Pyrolysis* **89**, 74-86, https://doi.org/10.1016/j.jaap.2010.05.009 (2010).

7 Hao, J. *et al.* TG-FTIR, Py-two-dimensional GC–MS with heart-cutting and LC–MS/MS to reveal hydrocyanic acid formation mechanisms during glycine pyrolysis. *J. Therm. Anal. Calorim.* **115**, 667-673, https://doi.org/10.1007/s10973-013-3214-0 (2014).

8 Leng, L. *et al.* Insights into glycine pyrolysis mechanisms: Integrated experimental and molecular dynamics/DFT simulation studies. *Fuel* **351**, 128949, https://doi.org/10.1016/j.fuel.2023.128949 (2023).

9 Li, J. *et al.* Evaluate the pyrolysis pathway of glycine and glycylglycine by TG–FTIR. *J. Anal. Appl. Pyrolysis* **80**, 247-253, https://doi.org/10.1016/j.jaap.2007.03.001 (2007).

10 Xu, Z.-X. *et al.* Investigation of pathways for transformation of N‑heterocycle compounds during sewage sludge pyrolysis process. *Fuel Process. Technol.* **182**, 37-44, https://doi.org/10.1016/j.fuproc.2018.10.020 (2018).

11 Chen, H. *et al.* Investigation on co-pyrolysis of lignocellulosic biomass and amino acids using TG-FTIR and Py-GC/MS. *Energy Convers. Manage.* **196**, 320-329, https://doi.org/10.1016/j.enconman.2019.06.010 (2019).

12 Jie, L. *et al.* The investigation of thermal decomposition pathways of phenylalanine and tyrosine by TG–FTIR. *Thermochim. Acta* **467**, 20-29, https://doi.org/10.1016/j.tca.2007.10.014 (2008).

13 Zhao, X.-Y., Jiang, W., Shan, Y.-F. & Cao, J.-P. Mechanism Study on Nitrogen Migration and Catalytic Denitrification during the Pyrolysis of Lysine and Tryptophan. *Energy Fuels* **36**, 502-513, https://doi.org/10.1021/acs.energyfuels.1c03818 (2022).

14 Choi, S.-S. & Ko, J.-E. Analysis of cyclic pyrolysis products formed from amino acid monomer. *J. Chromatogr.* **1218**, 8443-8455, https://doi.org/10.1016/j.chroma.2011.09.055 (2011).
